# What Epidemiologists Can Do in the Era of Machine Learning and Artificial Intelligence

**DOI:** 10.2188/jea.JE20240412

**Published:** 2025-06-05

**Authors:** Akihiro Nishi, Kosuke Inoue

**Affiliations:** 1 Department of Epidemiology, Fielding School of Public Health, University of California, Los Angeles, California, United States; 2 California Center for Population Research, University of California, Los Angeles, California, United States; 3 Department of Social Epidemiology, Graduate School of Medicine, Kyoto University, Kyoto, Japan; 4 Hakubi Center for Advanced Research, Kyoto University, Kyoto, Japan

To the Editor:

Epidemiologists have qualitatively and quantitatively examined and provided knowledge that helps understand the influence of risk or protective factors, ranging from genetic to socio-political factors, for health and illness outcome variables at the population level. In the 2020s, modern epidemiologists have witnessed the era of machine learning, deep learning, and, more broadly, artificial intelligence (AI), which emulates human intelligence using computers.^1^ Many epidemiologists have tried applying AI in their research. However, how epidemiologists can interact with AI has yet to be summarized or itemized holistically. Therefore, in the present letter, we brainstorm and discuss seven perspectives. Although some perspectives have already been hot research areas,^1^^,^^2^ many remain on the epidemiologists’ future to-do list.

First, AI-based prediction models can qualitatively indicate how epidemiologists should theorize the relationship of risk or protective factors for an outcome variable. For example, suppose epidemiologists initially theorize and hypothesize two factors independently cause an outcome (X_1_ → Y ← X_2_), and a more flexible AI-based prediction model exhibits a higher predictive ability than a conventional linear regression model. While the better prediction does not necessarily suggest its usefulness in causal modeling, it may encourage them to update their model to AI-based flexible models (eg, SuperLearner^2^), accounting for the role of a non-linear contribution and potential synergy of X_1_ and X_2_, which the conventional model might not incorporate initially. Such contribution can be observed in genetic and multi-omics epidemiology.^3^

Second, AI allows epidemiologists to quantify what was not quantifiable previously. For example, wearable devices have brought large spatiotemporal data; texts and images in electric health records can be convertible to analyzable data using natural language and image processing. Such new data types can help epidemiologists identify new risk or protective factors and detect diseases or illnesses earlier. For example, a previous study applied AI to electrocardiograms to build the prediction model for chemotherapy-induced cardiotoxicity.^4^ The other example is the application of deep learning to cardiac magnetic resonance images to assess the size of the ascending and descending thoracic aorta.^5^ Researchers then conducted genome-wide association studies and found a genetic basis for variations in the size of the ascending and descending thoracic aorta, which helps us better understand the disease processes underlying aneurysms and dissections.

Third, AI can enhance causal inference. For example, AI has been widely applied to causal modeling, such as propensity score matching, g-formula, and doubly robust estimators.^2^ Recently, AI has also contributed to estimating heterogeneous treatment effects, accounting for the complex interplay of multiple individual characteristics.^6^ Such an approach allows researchers to estimate the treatment effect for each individual based on their observed characteristics and target individuals who are estimated to have a large treatment effect (ie, “high-benefit approach”).^7^ Because conventional subgroup analyses were not able to assess such high-dimensional interaction, the AI application in this field has the potential to promote the discussion about personalized medicine and public health.

Fourth, in addition to conventional association/causation studies involving independent and dependent variables, epidemiologists also work in systems science, such as infectious disease modeling and prediction, where more dynamic and non-acyclic flows of multiple factors are assumed over time.^8^ For example, reinforcement learning was used to optimize the dynamic treatment strategies for adult patients with sepsis in the intensive care unit.^9^

Fifth, it is also important to note that AI can be a risk or protective factor for health and illnesses. Human-AI interactions, including using generative AIs (eg, ChatGPT and Gemini), may enhance or impair people’s health. For example, AI-driven care robots may enhance care recipients’ health by early detection of injury risks, while they may impair mental health by accelerating social isolation from other humans. Thus, AI, using it, and AI-human interactions can be social determinants of health.

Sixth, since it is known that AIs sometimes produce algorithmic unfairness (dependency of the model results on variables representing race, gender, and others),^1^^,^^10^ epidemiologists should be interested in monitoring the unintended negative consequences of the use of “black-box” AI-based models, especially among vulnerable populations. For example, AI-based prediction models trained by publicly available chest x-ray datasets incorrectly predict “no finding” for females and Black and Hispanic patients.^11^ To mitigate such a bias, bias-mitigated techniques (eg, *fairlearn* in Python and *aif360* [IBM’s AI Fairness 360] in R) can be used.^10^

Finally, AIs may help and redefine the work of epidemiologists in the future. For example, *Pcalg* is an AI package in R for causal discovery, which can deliver potential causal structures for provided data (constraint-based search methods) or assign a relevance score for causal diagrams (search-based methods).^12^ This implies that the theoretical work that epidemiologists have done—drawing causal diagrams based on their theory and prior knowledge—can be supported by AIs and data. Developing these AI-based epidemiologic methods is also included in the work of epidemiologists.
